# Robust Audio Content Classification Using Hybrid-Based SMD and Entropy-Based VAD

**DOI:** 10.3390/e22020183

**Published:** 2020-02-06

**Authors:** Kun-Ching Wang

**Affiliations:** Department of Information Technology & Communication, Shih Chien University, No. 200, University Rd, Neimen Shiang, Kaohsiung 845, Taiwan; kunching.wang@gmail.com

**Keywords:** audio content classification, spectral entropy, voice activity detection, speech/music discrimination, wavelet packet, support vector machine

## Abstract

A robust approach for the application of audio content classification (ACC) is proposed in this paper, especially in variable noise-level conditions. We know that speech, music, and background noise (also called silence) are usually mixed in the noisy audio signal. Based on the findings, we propose a hierarchical ACC approach consisting of three parts: voice activity detection (VAD), speech/music discrimination (SMD), and post-processing. First, entropy-based VAD is successfully used to segment input signal into noisy audio and noise even if variable-noise level is happening. The determinations of one-dimensional (1D)-subband energy information (1D-SEI) and 2D-textural image information (2D-TII) are then formed as a hybrid feature set. The hybrid-based SMD is achieved because the hybrid feature set is input into the classification of the support vector machine (SVM). Finally, a rule-based post-processing of segments is utilized to smoothly determine the output of the ACC system. The noisy audio is successfully classified into noise, speech, and music. Experimental results show that the hierarchical ACC system using hybrid feature-based SMD and entropy-based VAD is successfully evaluated against three available datasets and is comparable with existing methods even in a variable noise-level environment. In addition, our test results with the VAD scheme and hybrid features also shows that the proposed architecture increases the performance of audio content discrimination.

## 1. Introduction

With the rapid growth of information technology, multimedia management is a very crucial task. Multimedia is needed to classify different data types for efficient accessing and/or retrieving. Knowing how to build a management of multimedia information for AV (audio/video) indexing and retrieval is becoming extremely important. In the field of AV indexing and retrieval, the speech/music discrimination (SMD) is a very crucial task for the audio content classification (ACC) system or general audio detection and classification (GADC) [1,2,3,4,5,6,7,8,9,10,11,12,13,14,15,16,17,18]. In recently, the SMD literatures have been presented in different application [19,20,21,22,23,24] and closely related to retrieval of audio content indexing [20]. In general, audio feature extraction and audio segmentation are two main parts of a content-based classifier. Different features are presented to describe audio data. These features are mainly categories characteristic of time-domain and frequency-domain. In terms of feature extraction, the very common time-domain features are short-time energy (STE) [25,26] and the zero-crossing rate (ZCR) [27,28]. Signal energy [29,30,31], fundamental frequency [32], Mel frequency cepstral coefficients (MFCC) [19,33,34] are the most used frequency-domain features. Recently, a few studies focused on speech and song/music discrimination [35,36,37]. Some features such as loudness and sharpness have been incorporated in the human hearing process to describe sounds [38,39]. In a study by [40], a novel feature extraction method based on the visual signature extraction is presented. The well-known “spectrogram reading” is regarded as visual information and displays the representation of time-frequency. In the visual domain, the representation of time-frequency successfully stands for the audio signal pattern [40,41]. In addition, various techniques of audio classification are used for characterizing music signals, such as threshold-based methods or combining the string tokenization method and data mining technique [42]. Neural network [43], clustering [44], and k-nearest neighbor (k-NN) are used for speech/music classification, and the decision is made based on a heuristic-based approach [45]. In [46], the decision relies on the k-NN for classification by using perceptually weighted Euclidean distance. Gaussian mixture models (GMM) [47], support vector machine (SVM) [48], and fuzzy-rule [49] are also used for speech/music classification. Such new trends include temporal feature integration and classifiers aggregation [8,9,10,11,12,13,14,15], novelty audio detection and bimodal segmentation [7,9,16], and deep learning [5,17]. In recent years deep learning algorithms have been successfully used to solve numerous speech/noise classification problems, especially the development of deep convolutional neural networks without any need for careful feature selection [50,51]. However, deep neural networks are generally known to be more computationally expensive and slower than other more conventional methods [52]. Apart from the above, innovative techniques utilizing one-class classifiers, perceptual wavelet-cepstral parameters, hierarchical/multi-resolution thresholding, and other adaptive detection mechanisms were recently reported [7,9,10,11].

**Figure 1:**
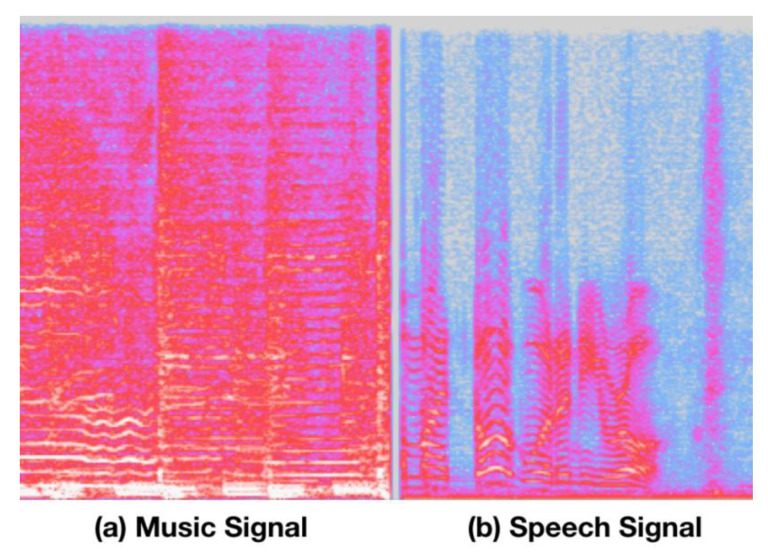
Sound spectrogram for music and speech. (**a**) Spectrogram on music and (**b**) spectrogram on speech.

Up to now, in a real-life environment, the problem of a variable-noise level environment is not considered for the above-mentioned works. To alleviate this problem, the robust spectral entropy-based scheme of voice-activity detection (VAD) which distinguishes speech and non-speech segments from the incoming audio signal is combined with the utilized SMD approach as a front-end of the proposed system of ACC application. Especially for the VAD case, the idea of using spectral entropy and other related parameters that monitor spectral variability or flatness has been used for many years [1,3,4]. Our previous research article [53] proved that spectral entropy-based VAD can be successfully applied to a variable noise-level environment. In addition, the differences on the sound spectrogram between music and speech are significant. In music, the spectrum’s peak tends to change relatively slowly even though music is played with various tempos as shown in Figure 1. On the contrary, shorter durations occur in speech sound events. We know that the spectral envelope of speech varies more frequently than the spectral envelope of music. Consequently, the rate of change of the spectral envelope (or called texture diversity) is one of the valid features for characterizing the differences between speech and music. This type of texture diversity suggests that perceptual wavelet analysis on a spectrogram will generate highly discriminate features for audio discrimination. Texture diversity is also regarded as 2D textual image information on a spectrogram and was successfully applied in studies by [54] and [55].

Extended from our previous work [56], a hierarchical scheme of the ACC system is proposed in this paper. In general, audio hierarchically categorizes silence/background noise, various music genres, and speech signals. As a result, a three-stage scheme involving speech, music, and other is adopted herein [9]. In the first stage of the proposed ACC, the incoming audio signal is pre-emphasized and partitioned. Next, the scheme of VAD is utilized with the Mel-scale spectral entropy to classify the emphasized audio signal into silence segments and non-silence segments. In the second stage, the SMD approach comprises of the extraction of hybrid features and SVM-based classification. A novel technique of hybrid feature extraction is derived from wavelet-spectrogram textual information and energy information to obtain a set of features including the 1D subband energy information (1D-SEI) and 2D texture image information (2D-TII).

In order to extract the 2D-TII parameter, we first generated the spectrogram in grayscale. Then, the local information was captured by zoning the range from 0 kHz to 4 kHz in order to characterize the discrimination between speech and music [57]. This is so the 2D-texture information [54] can be analyzed upon the wavelet-spectrogram. Next, the 2D-TII parameter is accurately obtained by using Laws’ mask through 2D-perceptual wavelet packet transform (PWPT). Consequently, we let three hybrid feature inputs into an SVM classifier. During the second stage, the noisy audio segments are classified into speech segments and music segments. In the third stage we improved the discrimination accuracy, and a rule-based post-processing method was applied to reflect the continuity of audio data in time.

This paper is organized as follows. In Section 2, we introduce the proposed approach of the three-stage ACC. The approach includes three main stages: pre-processing/VAD, SMD, and post-processing. The VAD uses the measure of band-spectral entropy to distinguish non-noise segments (noisy audio segment) from noise segments (silence). Section 3 presents the hybrid-based SMD algorithm. The hybrid features include 1D subband energy information (1D-SEI) and 2D texture image information (2D-TII). Through the combination of 1D signal processing and 2D image processing, the hybrid features characterize the discrimination between speech and music. In Section 4, the rule-based post-processing is presented to improve the segmentation results in different noise types and levels. Finally, the experiments and results are presented in Section 5. In this section, the evaluation of the proposed ACC approach is performed on well-known speech and music databases (e.g., GTZAN dataset) at well-defined signal-to-noise ratio (SNR) levels. Section 6 provides the discussion and conclusions.

## 2. The Architecture of Hierarchical Based ACC Approach

**Figure 2:**
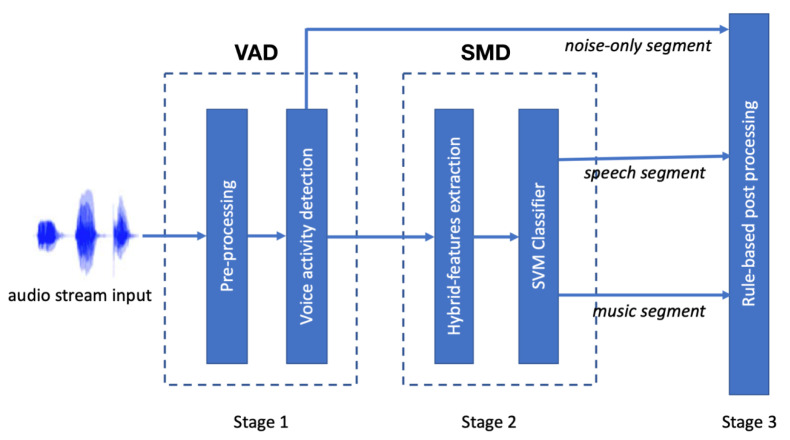
The flowchart for deriving the proposed hierarchical audio content classification with entropy-based voice activity detection (VAD), hybrid-based speech/music discrimination (SMD), and rule-based post-processing. SVM = support vector machine.

Figure 2 shows the block diagram of the audio content classification (ACC) system, which is divided into three main stages: pre-processing/voice activity detection (VAD), speech/music discrimination (SMD), and rule-based post-processing. The details are described below.

### 2.1. Pre-Processing

In order to emphasize the important higher-frequency elements, the speech signal is first high-pass filtered. The speech frame, x[n], is then divided into several segments. Those segments are chosen as frame size = 256 samples and 50% overlapping with the neighboring frame. The Hamming window is applied to each segment after frame partitioning [58].

### 2.2. Spectral Entropy-Based Voice Activity Detection (VAD)

The VAD differs from speech/music discrimination (SMD). VAD discriminates between noise and speech while SMD discriminates between speech and music [1,2,3,4,5,6,7].

The conventional VAD algorithms rely on short-time energy or spectral energy as the primary feature parameters with the augmentation of zero-crossing rate, pitch, and duration information [59,60]; yet these features become less reliable in the presence of non-stationary noise and various types of sound artifacts. Extended from previous works [53,61], a spectral entropy-based voice activity detection (VAD) scheme was successfully used for segmenting the noisy signal into noise-only segments and noisy audio segments especially for variable noise-level. Herein, the spectral entropy-based VAD is utilized in the first stage of the ACC system.

In a previous work [61], the band-partitioning spectral entropy (BSE) parameter, $H_{BSE}$, was presented as follows:

(1) $$H_{BSE}=\sum_{m=1}^{N_{b}}W\left(m\right)\cdot P\left(m\right)\cdot log\left[1/P\left(m\right)\right]$$

where $N_{b}$ is the total band size of each frame ($N_{b}=32$ uniform-bands). W(m) indicates the weight of the $m^{th}\;$band defined as follows:

(2) $$W\left(m\right)=var\left[\min\left(P_{b}\right)/P_{b}\left(m-1\right),P_{b}\left(m\right),P_{b}\left(m+1\right)\right]$$

where var(·) represents the variance. $P_{b}\left(m\right)$ represents the probability associated with band energy described as follows:

(3) $$\begin{array}{cc} P_{b}\left(m\right)=E_{b}\left(m\right)/\sum_{k=1}^{N_{b}}E_{b}\left(k\right), & 1\leq m\leq N_{b} \end{array}$$

where $E_{b}\left(m\right)$ represents the band energy of the $m^{th}$ band.

**Figure 3:**
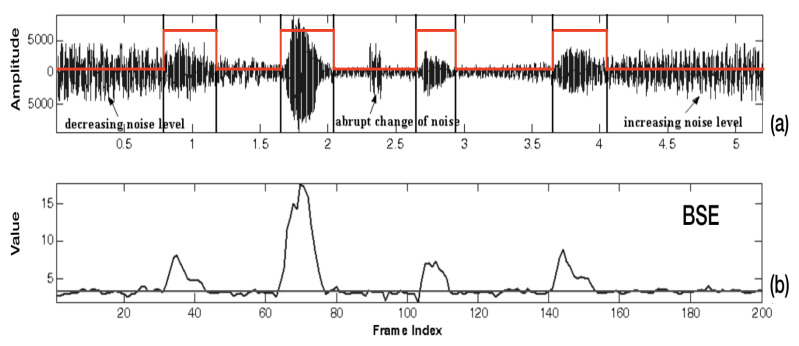
The measurement of spectral entropy is robust against changing signal levels even though the amplitude of background noise varies with the environmental state. (**a**) The audio signal mixed with variable-noise level. (**b**) The value of band-partitioning spectral entropy (BSE) parameter over a period of time.

Figure 3 shows that the measurement of spectral entropy is robust against changing signal levels even though the amplitude of background noise varies with the environmental state because the spectral entropy depends only on the variation of spectral energy, but not on the amount of spectral energy.

## 3. Hybrid-Based Speech/Music Discrimination (SMD)

**Figure 4:**
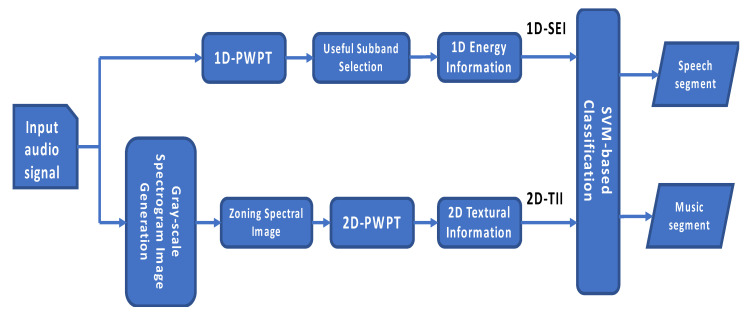
The flowchart of the hybrid-based speech/music discrimination. 1D/2D-PWPT = one/two-dimensional perceptual wavelet packet transform.

The processing flow of the hybrid-based SMD is shown in Figure 4. The SMD is based on a hybrid feature set, which contains 1D subband energy information (1D-SEI) and 2D texture information (2D-TII) parameters. For noisy segmented audio input, the composed features are extracted from the 1D-PWPT and Bark scale spectrogram image, respectively. The hybrid features include 1D-SEI feature set and 2D-TII feature set. For the feature extraction of 1D-SEI, we used 1D-PWPT (perceptual wavelet packet transform) to get 24 critical subbands. Through the useful subband selection, the correct energy information was used to discriminate the difference between speech and music. In the feature extraction of 2D-TII, gray-scale spectrogram was first generated. Zoning the range from 0 kHz to 4 kHz, the local information is enough to characterize speech and music, respectively. Using 2D-PWPT, we can get the 2D textural information. Finally, the hybrid features are then fed into the SVM-based classifier to discriminate their types (speech or music).

### 3.1. D-PWPT (Perceptual Wavelet Packet Transform)

In order to mimic the hearing characteristics of human cochlea, the Bark scale, a psychoacoustical scale proposed by Eberhard Zwicker in 1961, was used [62]. It was found that for the auditory quality of a speech signal, an analysis on non-uniform frequency resolution is better than on uniformly spaced frequency resolution [63]. In fact, the selection of the “optimal” decomposition is a classical problem in order to suppress audible noise and eliminate audible artefacts. According to the Bark scale rules, the 1D-perceptual wavelet packet transform (PWPT) implemented with an efficient five-stage tree structure is utilized to split 24 critical subbands for input speech signal. For each stage, the high-pass filter and low-pass filter are implemented with the Daubechies family wavelet, where the symbol ↓2 denotes an operator of down-sampling by 2 [53]. In Table 1, we see that the Bark scale-based wavelet decomposition lets every frequency band limit become more and more linear when frequencies are below 500 Hz; this scale is more or less equal to a logarithmic frequency axis when above about 500 Hz.

### 3.2. Optimal Subband Selection for Useful Information

In previous works [64], an extraction of selecting useful frequency subbands was proposed to suppress the noise effect on the ACC system, especially at a poor SNR (signal-to-noise ratio). The process of pure energy on the useful frequency is shown below.

During the initialization period, the noisy signal was assumed to be noise-only, and the noise spectrum was estimated by averaging the initial 10 frames. To recursively estimate the noise power spectrum, the subband noise power, $\tilde{N}\left(\zeta ,m\right)$, was adaptively estimated by smoothing filtering.

For the $m^{th}\;$frame, the spectral energy of the $\zeta^{th}$ subband is evaluated by the sum of squares:

(4) $$E\left(\zeta ,m\right)=\sum_{\omega_{\zeta ,l}}^{\omega_{\zeta ,h}}{\left|w\left(\omega ,m\right)\right|}^{2}$$

where w(ω,m) means the $\omega^{th}$ wavelet coefficient. $\omega_{\zeta ,l}$ and $\omega_{\zeta ,h}$ denote the lower boundaries and the upper boundaries of the $\zeta^{th}$ subband, respectively.

The $\zeta^{th}$ frequency subbands energy of pure speech signal of the $m^{th}\;$frame $\tilde{E}\left(\zeta ,m\right)$ is estimated:

(5) $$\tilde{E}\left(\zeta ,m\right)=E\left(\zeta ,m\right)-\tilde{N}\left(\zeta ,m\right)$$

where  N ˜(ζ,m) is the noise power of the $\zeta^{th}$ frequency subband.

According to Wu et al. [65], subbands with a higher energy $\tilde{E}\left(\zeta ,m\right)\;$can stand for a greater amount of pure speech information. So, the frequency subband should be sorted according to its value of $\tilde{E}\left(\zeta ,m\right)$.

That is,

(6) $$E\left(I_{1,}m\right)\geq E\left(I_{2,}m\right)\geq \cdots \geq E\left(I_{N_{ub}\left(m\right),}m\right),I\in \left[I_{1},I_{2},I_{3},\cdots ,I_{N_{ub}\left(m\right)}\right]$$

where $I_{i}$ is the index of the frequency subband with the $i^{th}$ max energy. $N_{ub}\left(m\right)$ denotes the number of useful subbands on the $m^{th}$ frame. $I\in \left[I_{1},I_{2},I_{3},\cdots ,I_{N_{ub}\left(m\right)}\right]$.

**Figure 5:**
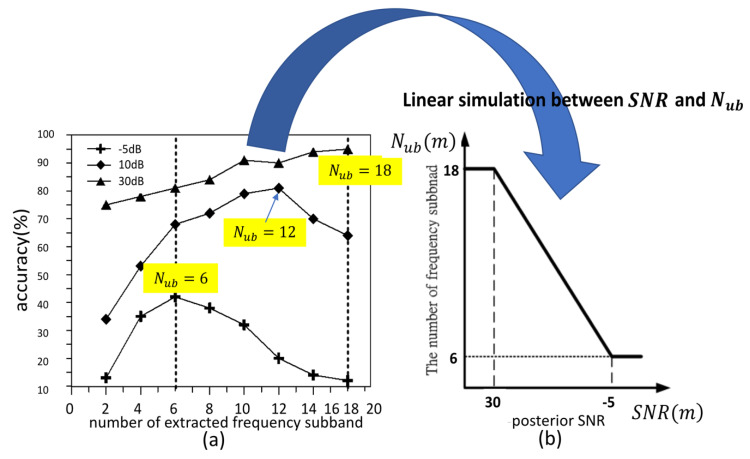
The relation between the number of useful frequency subbands, $N_{ub}\left(m\right)$, and the posterior signal-to-noise ratio (SNR), SNR(m). (**a**) The results of correct detection accuracy with different frequency subbands at −5 dB, 10 dB, and 30 dB. (**b**) A linear function of the relationship between $N_{ub}\left(m\right)$ and SNR(m).

In fact, the relation between the number of useful frequency subbands, $N_{ub}\left(m\right)$, and the posterior SNR, SNR(m), has a negative-correlation, as shown in Figure 5.

We see that the number of useful frequency subbands increases with the increase of SNR in Figure 5a. When SNR(m)=−5, SNR(m)=10, and SNR(m)=30dB, the highest accuracy of VAD appears as $N_{ub}\left(m\right)=6$, $N_{ub}\left(m\right)=12,$ and $N_{ub}\left(m\right)=18$, respectively. In order to simulate the relationship between $N_{ub}\left(m\right)$ and SNR(m), a linear function is in the boundary between −5 dB and 30 dB, while the duration between $N_{ub}\left(m\right)=6$ to $N_{ub}\left(m\right)=18$ is shown in Figure 5b:

(7) $$N_{ub}\left(m\right)=\{\begin{array}{cc} 6 & ,SNR\left(m\right)<-5\;\mathrm{dB}\\ \left[\left(18-6\right)\times \frac{\left(SNR\left(m\right)-\left(-5\right)\right)}{30-\left(-5\right)}+6\right] & ,-5\;\mathrm{dB}\leq SNR\left(m\right)\leq 30\;\mathrm{dB}\\ 18 & ,SNR\left(m\right)>30\;\mathrm{dB} \end{array}$$

where [·] is the round off operator and SNR(m) denotes a frame-based posterior SNR for the $m^{th}\;$frame. SNR(m) is dependent on the summation of subband-based posterior SNR snr(ζ,m) on the $\zeta^{th}$ useful subband, defined as:

(8) $$SNR\left(m\right)=10\times log_{10}\sum_{\zeta \in N_{ub}}snr\left(\zeta ,m\right),$$

where $snr\left(\zeta ,m\right)={\left|E\left(\zeta ,m\right)\right|}^{2}/\tilde{N}\left(\zeta ,m\right)$.

**Figure 6:**
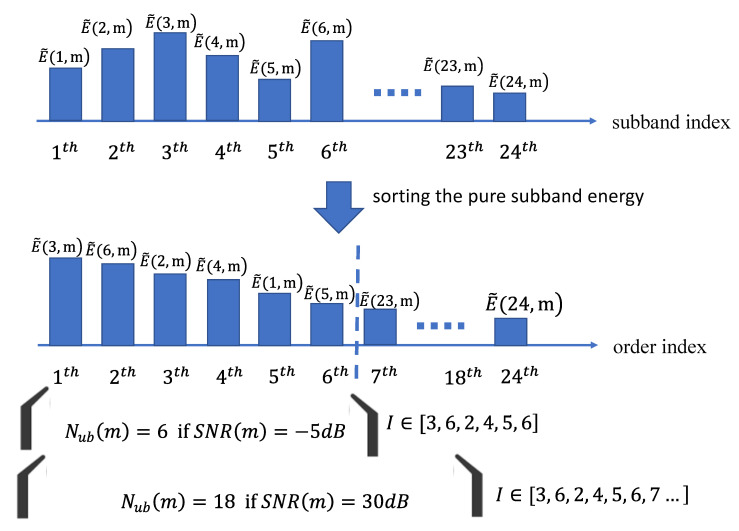
Example for extracting the useful subband index and number according to the value of the posterior SNR.

Figure 6 clearly illustrates the example of extracting useful subbands under a different posterior SNR. We see that the pure subband energy is rearranged after sorting processing among all 24 subbands. Originally, the first subband index ζ is 1, but the updated first index ζ is 3 when sorting the energy. Consequently, the useful subband index and number are extracted according to the value of the posterior SNR.

### 3.3. The 1D Subband Energy Informations (1D-SEIs)

It is well-known that the distribution of energy on each frequency band is a very relevant acoustic cue. After selecting a useful subband, the wavelet energy was calculated from 1D-PWPT to form a 1D subband energy informations (1D-SEIs): the average of subband energy (ASE), the standard deviation of subband energy (SDSE), and Teager energy. So, the 1D-SEIs derived from three parameters are investigated below:

-- The average of subband energy (ASE)
  (9) $$E^{avg}\left(m\right)=\sum_{1}^{N_{ub}\left(m\right)}\tilde{E}\left(\zeta ,m\right)/N_{ub}\left(m\right),\;\zeta \in \left[I_{1},I_{2},I_{3},\cdots ,I_{N_{ub}\left(m\right)}\right].\;$$
-- The standard deviation of subband energy (SDSE)
  (10) $$E^{sd}\left(m\right)=1+\sqrt{\frac{1}{24}\sum_{\zeta =1}^{24}{\left(\tilde{E}\left(\zeta ,m\right)-E^{avg}\left(m\right)\right)}^{2}}.$$
  We see that the speech’s energy exists in a lower frequency band mainly and the music’s energy is in a wide range of the frequency band.
-- Teager energy
  (11) $$E^{teg}\left(m\right)=\tilde{E}{\left(\zeta ,m\right)}^{2}-\tilde{E}\left(\zeta ,m-1\right)\times \tilde{E}\left(\zeta ,m+1\right).$$

The discrete Teager energy operator (TEO), introduced by Kaiser [66], allows modulation energy tracking and gives a better representation of the formant information in the feature vector. So, we can also successfully use the characteristic to discriminate speech from music.

### 3.4. Gray-Scale Spectrogram Image Generation

In this subsection, a novel feature extraction is derived from the gray-scale spectrogram images. As mentioned above, we see the difference between speech and music while relying on the virtual representation of audio data by spectrogram. In fact, the gray-scale spectrogram images are regarded as a time-frequency-intensity representation. Since the human perception of sound is logarithmic, the log-spectrogram is defined as:

(12) $$S_{log}\left(k,t\right)=log\left(\left|X\left(k,t\right)\right|\right).$$

The time-frequency-intensity representation is normalized into a grayscale normalized image, within the range of 0 to 1:

(13) $$R_{SpecImg}\left(k,t\right)=\left(S_{log}\left(k,t\right)-S_{min}\right)/\left(S_{max}-S_{min}\right).$$

### 3.5. The Zoning for Spectrogram Image 

To achieve good results for SMD, the zoning method for spectrogram image was applied [67]. In fact, the textural image information between speech signals and music data is different [68]. It was found that the music audio data consist of a few silent intervals, and have continuous energy peaks for a short time and fewer frequency variations, while the speech audio data consist of many silent intervals and most of the energy is located at the lower frequencies [69]. Accordingly, the spectrogram image from 0 kHz to 4 kHz is separated to extract textural features as local features by the zoning method. The feature extraction for the 2D textural image information (2D-TII) is discussed in the next subsection.

### 3.6. The 2D Textural Image Information (2D-TII)

In fact, the differences on the sound spectrogram between music and speech are significant. In music, the spectrum’s peak tends to change relatively slowly even though the music is played with various tempos. On the contrary, in speech, sound events often have shorter durations but with more distinctive time-frequency representations. For the above reason, the 2D-TII features can be successfully derived from the audio spectrogram image through Laws’ masks based on the principle of texture energy measurement [54] to find the difference between speech and music. It is known that Laws’ masks are well described for texture energy variation in image processing, and the masks consist of five masks derived from one-dimensional vectors, such as edge $E_{5}$, level $L_{5}$, spot $S_{5}$, ripple $R_{5},$ and wave $W_{5}$ expressed as Equations (14)–(18):

(14) $$E_{5}=Edge\;detection:\;\left[\begin{array}{ccc} -1 & -2 & \begin{array}{cc} 0 & \begin{array}{cc} 2 & 1 \end{array} \end{array} \end{array}\right]$$

(15) $$L_{5}=Level\;detection:\;\left[\begin{array}{ccc} 1 & 4 & \begin{array}{cc} 6 & \begin{array}{cc} 4 & 1 \end{array} \end{array} \end{array}\right]\;$$

(16) $$S_{5}=Spot\;detection:\;\left[\begin{array}{ccc} -1 & 0 & \begin{array}{cc} 2 & \begin{array}{cc} 0 & -1 \end{array} \end{array} \end{array}\right]\;$$

(17) $$R_{5}=Ripple\;detection:\;\left[\begin{array}{ccc} 1 & -4 & \begin{array}{cc} 6 & \begin{array}{cc} -4 & 1 \end{array} \end{array} \end{array}\right]\;$$

(18) $$W_{5}=Wave\;detection:\;\left[\begin{array}{ccc} -1 & 2 & \begin{array}{cc} 0 & \begin{array}{cc} -2 & 1 \end{array} \end{array} \end{array}\right]\;$$

The two-dimensional filters of the size 5 × 5 were generated by convoluting any vertical one-dimensional vector with a horizontal one. Finally, the 25 combinations of two-dimensional masks are determined [70].

First, we convoluted the image with each two-dimensional mask to extract texture information from an image $I_{\left(i,j\right)}$ of size (M×N). For example, if we used $E_{5}E_{5}$ to filter the image $I_{\left(i,j\right)}$, the result was a texture image, $TI_{E_{5}E_{5}}$, as seen in Equation (19).

(19) $$TI_{E_{5}E_{5}}=I_{i,j}⊗E_{5}E_{5}$$

All the two-dimensional masks, except $L_{5}L_{5}$, had a zero mean. According to Laws, texture image $TI_{L_{5}L_{5}}$ was used to normalize the contrast of all the texture images $TI_{\left(i,j\right)}$, as seen in Equation (20).

(20) $$Normalize\left(TI_{mask}\right)=TI_{mask}/TI_{L_{5}L_{5}}.$$

Next, the outputs (TI) from Laws’ masks were passed to “texture energy measurement” (TEM) filters. We calculate the non-linear interval by processing TI normalized and yield through “Texture Energy Measurements, (TEM)” filter. This consisted of a moving non-linear window average of absolute values, as seen in Equation (21).

(21) $$TEM_{ij}=\sum_{u=-7}^{u=7}\sum_{v=-7}^{v=7}\left[Normalize\left(TI_{i+u,j+v}\right)\right].$$

Since not all mask energy is used as the input basis of texture energy, we take out unchangeable TR values before and after rotation to obtain a valid TEM. The TR derived from TEM is represented in Equation (22).

(22) $$TR_{E5L5}=\left(TEM_{E5L5}+TEM_{L5E5}\right)/2$$

After Equation (22), the results of the three texture feature values: mean, standard deviance (SD), and entropy are extracted via Equations (23)–(25) to exploit the variation of texture information.

(23) $$Mean=\sum_{i=0}^{M}\sum_{j=0}^{N}TR_{ij}/\left(M\times N\right),$$

(24) $$\;SD=\sqrt{\sum_{i=0}^{M}\sum_{j=0}^{N}{\left(TR_{ij}-Mean\right)}^{2}/\left(M\times N\right)},$$

(25) $$Entropy=\sum_{i=0}^{M}\sum_{j=0}^{N}TR_{ij}{}^{2}/\left(M\times N\right).$$

Each equation produces feature vectors with 14-dimensional size. Finally, a total of three feature vectors with 42-dimensional sizes are used as the input data for training the SVM classifier.

### 3.7. From 2D-PWPT to 2D-TII 

**Figure 7:**
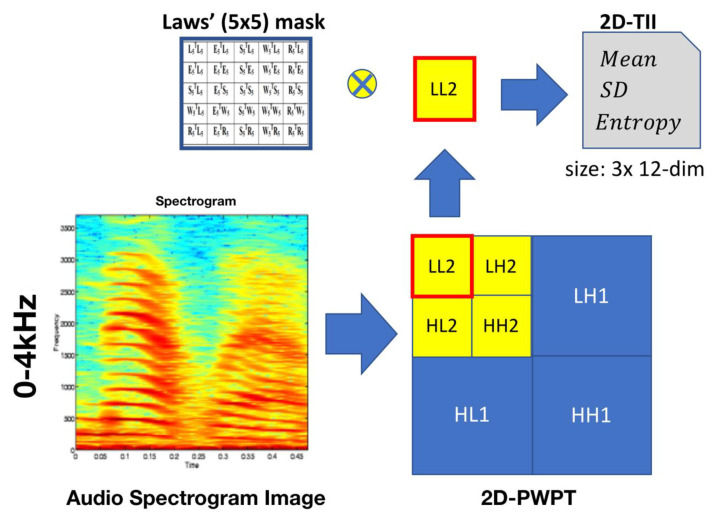
The block-diagram of the two-dimensional textural image information (2D-TII) derived from LL2 sub-image.

To perform texture analysis on multi-resolution, 2D-PWPT is utilized into an audio spectrogram image, which ranges from 0 to 4 KHz. Figure 7 shows an audio spectrogram image decomposition. In Figure 7, these subbands are first obtained using one-level wavelet decomposition. These subbands are labeled as LH1, HL1, and HH1 and represent the detail images, while the sub-band labeled as LL1 is regarded as the approximation image. The detail images represent the finest scale wavelet coefficients. Conversely, the approximation image corresponds to coarse level coefficients. The sub-band LL1 alone is further decomposed and critically sampled in order to obtain the next coarse level of wavelet coefficients. So, this results in two-level wavelet decomposition. Similarly, LL2 is used to obtain further decomposition. Lastly, the spectrogram image of LL2 is only convoluted by the two-dimensional Laws’ mask to determine the 2D-TII. Compared to the original image size of the spectrogram within 0 to 4 kHz, the LL2 is de-sized. Thus, we can decrease the computing time and get good information derived from LL2 sub-image that is better than the original image.

### 3.8. SVM-Based Classification

Support vector machine (SVM) is well-known effective bi-classification [71,72,73]. In actuality, the SVM is better than other conventional classifiers in terms of classification accuracy, computational time, and stability. In this subsection, the hybrid feature set including 1D-subband energy information and 2D-texture information, $F^{hyb}=\left[1D\_\mathrm{SEI},\;2D\_\mathrm{TII}\right]$, are imported into a discriminative classifier of the SVM to classify either the speech segment or music segment. Suppose a set $S=\left\{\left(x_{1},y_{1}\right),\ldots ,\left(x_{N},y_{N}\right)\right\}$of $R^{n}$ is the training set, where $x_{i}$ is the input signal vector, $y_{i}$ is the class label for speech or audio, $y_{i}\in \left\{-1,\;1\right\}$, and $R^{n}$ denotes n-dimensional space.

To find the optimal hyper-plane, the support vectors of the dataset maximize the margin, which is the distance between the hyper-plane and support vectors as follows:

(26) $$\;\min\;\frac{1}{2}{\left\|w\right\|}^{2}\;s.t.\;y_{i}\left(w^{T}x_{i}+b\right)\geq 1$$

The solution to the optimization problem of SVM is given by the Lagrange function as follows:

(27) $$L\left(\alpha \right)=\sum_{i=1}^{N}\alpha_{i}-\frac{1}{2}\sum_{i=1}^{N}\sum_{j=1}^{N}\alpha_{i}\alpha_{j}y_{i}y_{j}K\left(x_{i},x_{j}\right)$$

with constraint $\sum_{i=1}^{N}\alpha_{i}y_{i}=0$ and $0\leq \alpha_{i}\leq C$, where C is upper bound of the Lagrange multipliers $\alpha_{i}$ and the constant C∈[0, 1].

As for the kernel function, we consider ERBF and Gaussian function as shown below:

(28) $$K_{\mathrm{ERBF}}\left(x,\;y\right)=exp\left(-\gamma \left|x-y\right|/2\sigma^{2}\right),$$

(29) $$K_{\mathrm{Gaussian}}\left(x,\;y\right)=exp\left(-\gamma {\left|x-y\right|}^{2}/2\sigma^{2}\right),$$

where $\sigma^{2}$ is the variance. γ is the additional control parameter.

Potentially, the ERBF function is usually used as the kernel function and vastly improves the results [74]. Therefore, the SVM which adopts ERBF as a kernel function will be compared to other classification.

## 4. The Rule-Based Post-Processing

The purpose of the post-processing step is to reduce possible errors of segmentation and classification. The errors of segmentation may even be occurred due to abrupt changes in noise level. Here are some examples of rule-based schemes used in the post-processing step: if a “music” segment appears separately in a series of speech segments, it merges into that speech segment; if a “speech” segment appears separately in a series of music segments, it merges into that music segment; if a “music” segment appears in only two frames or is smaller than two frames, it merges into speech segments. The kernel of a rule-based engine is regarded as a set of IF-THEN rules. The formulations of a rule-based engine where speech is ‘S”, music is “M’, noise/silence/other is “N’, and “_” is represented as any audio type except for noise can be shown below:

(30) $$\begin{array}{l} R1:\mathrm{IF}\;N\_N\;\mathrm{THEN}\;NNN\\ R2:\mathrm{IF}\;SSMSS\;\mathrm{THEN}\;SSSSS\\ R3:\mathrm{IF}\;MMSMM\;\mathrm{THEN}\;MMMMM\\ R4:\mathrm{IF}\;MMSSS\;\mathrm{THEN}\;NSSSS\\ R5:\mathrm{IF}\;SSSMN\;\mathrm{THEN}\;SSSSN\\ R6:\mathrm{IF}\;NN\_NN\;\mathrm{THEN}\;NNNNNN\\ R7:\mathrm{IF}\;SSMMSSS\;\mathrm{THEN}\;SSSSSSS\\ R8:\mathrm{IF}\;SSSMMSS\;\mathrm{THEN}\;SSSSSSS\\ R9:\mathrm{IF}\;NNMMSSS\;\mathrm{THEN}\;NNSSSSS\\ R10:\mathrm{IF}\;SSSMMNN\;\mathrm{THEN}\;SSSSSNN \end{array}$$

**Figure 8:**
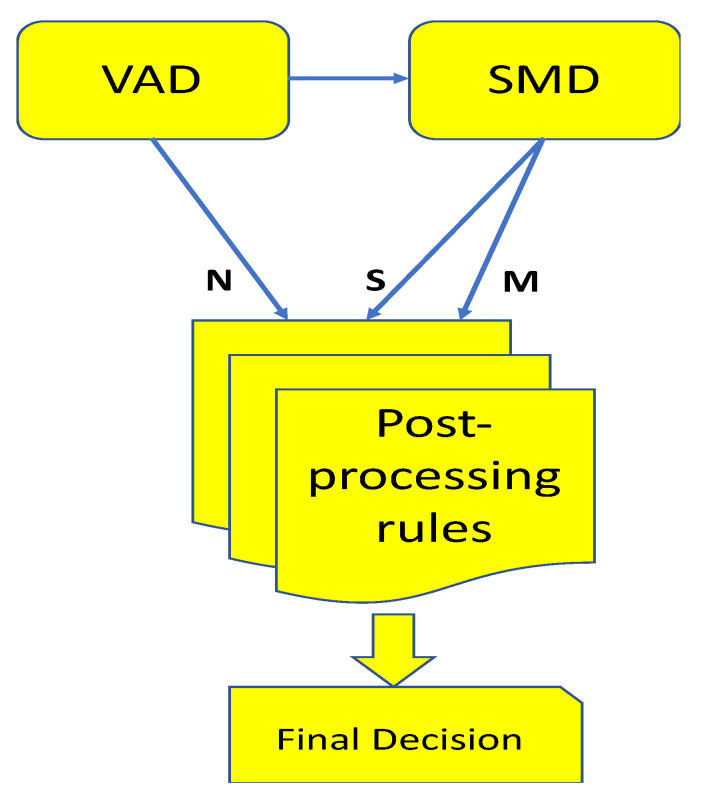
The block diagram of rule-based post-processing.

According to R1 to R10 from Equation (30), the procedure of a rule-based post-processing is fulfilled by the smoothing task as shown in Figure 8. Observing the figure, the hybrid features from SVM and VAD are regarded as input. After a complete loop over all the rules, the loop is repeated, until the segmentation remains unchanged.

## 5. Experimental Results

### 5.1. Database Description

To evaluate the proposed algorithm, the database consisted of three different subsets shown in Table 2. The first one is the well-known Music-Speech GTZAN library [75], which includes 120 tracks, each lasting 30 s and containing 60 examples of each class (music/speech). The second one is artificial audio data, which are artificially created by concatenating silence, speech, or music segments. The last one is real broadcasting recordings, which were collected from BBC radio, NHK, and TTV news. Subsets #2 and #3 were collected and organized from artificial audio data and real broadcasting recordings. The speech data come from news programs on the radio and TV stations, talks, as well as dialogs in movies, and the languages involve English, Chinese, and Japanese. In addition, the music consists of instrumental music and songs obtained from music CDs covering classic, pop, folk, and rock. The audio data stream was sampled in 16-bit with 8 kHz. In addition, many publicly available audio datasets including LVLib-SMO, FMA-small, and RWC Music Database are also introduced in Subsets #4, #5, and #6.

#### 5.1.1. Artificial Audio Data

Three test files were artificially created by concatenating silence, speech, or music segments. The speech signals spoken by a variety of both male and female speakers were taken from 12 to 15 min. The composition of the data set is shown as follows:

- Arti Num. 1: This is 15 min audio stream with alternate speech, music, and silence segments of equal (30 s) duration. This data set includes 12.54% of silence, 42.78% of speech, and 44.68% of music.
- Arti Num. 2: This data set also consists of 15 min audio stream comprising mainly of music data. In this case, 20 segments of music data are interleaved with shorter segments of speech. Therefore, this data set is composed of 9.36% of silence, 22.57% of speech, and 68.07% of music.
- Arti Num. 3: This data set also consists of 15 min audio stream comprising mainly of speech data. In this case, 20 s segments of music data are interleaved with shorter segments of speech. Therefore, this data set is composed of 11.58% of silence, 64.38% of speech, and 24.04% of music.

The results of three artificial files with different combinations of the sounds are shown in Table 3.

#### 5.1.2. Real Radio Recordings

The real TV news and real movie clips were recorded from BBC radio, NHK, and TTV news in order to evaluate the results of the proposed algorithm under realistic noisy environments. The durations of two real recording files range from 10 to 15 min where the length of the silence segment varies from 1 s to 3 s and the length of speech or music segments vary from 3 s to 10 s. To evaluate whether the proposed ACC algorithm is valid for a realistic environment, Table 4 shows the real TV news and real movie clips selected as Real #01 and Real #02, respectively.

### 5.2. Evaluation Results of Entropy-Based VAD

**Figure 9:**
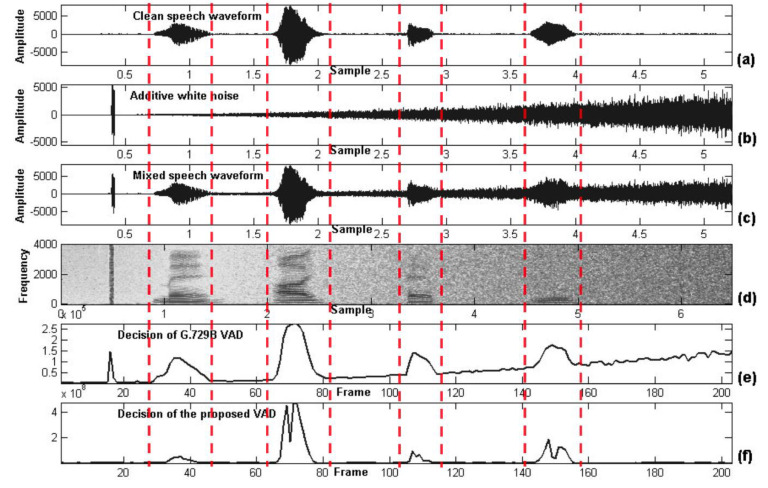
Comparison between the proposed VAD and G.729B. (**a**) Waveform of a clean speech ‘May I help you?’. (**b**) Waveform of an additive factory noise. (**c**) Waveform of a mixed speech signal. (**d**) Spectrogram of a mixed speech signal. (**e**) Result of G.729B VAD. (**f**) Result of the proposed VAD.

In the first evaluation, the experimental results of the entropy-based VAD is presented. The goal of VAD segmentation is to divide the audio signal into a voice segment (including speech and music) and non-voiced segment (including noise and background silence). Figure 9 shows segmentation performance of the entropy-based VAD proposed in our earlier work [53,61] against any variable noise-level conditions when comparing to the conventional method. It is found that the entropy-based parameter is related only to the variation of spectral energy but not to the amount of spectral energy, so the entropy-based algorithm outperforms the energy-based algorithm, especially in changing the level of noise.

To further evaluate the performance of the proposed VAD method, the following metrics were considered:

(31) $$Accuracy\left[\%\right]=\frac{TP+TN}{TP+FP+TN+FN}$$

(32) $$Precision\left[\%\right]=\frac{TP}{TP+FP}$$

(33) $$Recall\left[\%\right]=\frac{TP}{TP+FN}$$

(34) $$F1-score\left[\%\right]=\frac{2}{\frac{1}{Precision}+\frac{1}{Recall}}$$

where  TP, FP, TN, and FN represent true positive, false positive, true negative, and false negative rates, respectively. Accuracy[%] denotes the ratio correctly classified as voiced and non-voiced segments. Precision[%] indicates the ratio correctly classified as voiced segments when predicting a voiced segment. Recall[%] means the true positive rate when predicting a voiced segment and non-voiced segment. F1−score[%] is derived by calculation of the harmonic mean value between Precision[%] and Recall[%] . To examine the effectiveness of the entropy-based VAD, we compared it with other existing VAD methods. Table 5 shows the performance of the proposed VAD in terms of Acc[%], Pre[%], Rec[%], and F1[%] averaged over the SNR range from −5 dB to 30 dB testing on artificial audio data mixed background noise. In terms of accuracy, we found that the proposed entropy-based algorithm obtains the best Acc[%] with 90.04%. However, accuracy alone is not enough, and it is usually supplemented by Precision[%], Recall[%], and F1−score[%]. The results clearly show that the proposed entropy-based VAD is almost superior to other compared VADs.

### 5.3. The Evaluation of Hybrid-Based SMD

In actually, the Mel-frequency cepstral coefficient (MFCC), zero crossing (ZC), and spectral centroid (SC) are usually used as important features which are applied in the speech/music discrimination systems. In order to present the justification of the proposed hybrid features for speech/music discrimination, a comparison with other commonly used features is tested on data subsets #1, #2, and #3 and shown in Table 6. We see that the proposed hybrid feature set is superior to several well-known feature sets in terms of overall accuracy. The classification accuracy with 96.56% is the highest value while using a hybrid feature set.

In order to evaluate the performance of hybrid-based speech/music discrimination systems using a SVM classifier with ERBF kernel, the three different statistics are used below:

The percentage of true speech segments identified as speech,

(35) $$Speech\left(\%\right)=\frac{Correctly\;Specified\;Speech\;Segments}{Total\;Speech\;Segments}\;$$

The percentage of true music segments identified as music,

(36) $$Music\left(\%\right)=\frac{Correctly\;Specified\;Music\;Segments}{Total\;Music\;Segments}\;$$

The average percentage of correctly identified

(37) $$Average\left(\%\right)=\frac{Correctly\;Specified\;Segments}{Total\;Segments}\;$$

Table 7 shows the performance of hybrid-based SMD approach compared to various classifiers on GTZAN dataset. We can see from the results that the SVM classifier with ERBF kernel implies a better performance of the speech/music discriminator. The proposed hybrid-based approach provides the accuracy in Music as 90.41% due to that it gives rise to an important decrease of the MSE errors. On the contrary, the proposed hybrid-based approach achieves a highest average accuracy rate of 91.33% (the accuracy in Speech as 92.26% and in Music as 90.41%) among other classifiers.

### 5.4. The Robustness Evaluation of ACC System

In this subsection, the robustness performance of the overall system of audio content classification (ACC) is evaluated against any variable noise-level conditions. Combined with VAD scheme and the hybrid-based SMD, the hierarchical architecture of audio content classification (ACC) can provide higher performance. First, in order to perform the cross-validation evaluation on the proposed ACC, mismatched training and testing data are required. A case for the model testing is on BBC radio and NHK/TTV news and training is on the well-known GTZAN database. Table 8 shows that the evaluation of cross-validation is tested on the on BBC radio and NHK/TTV news database when the models are trained on the GTZAN database. It was found that the proposed ACC system can successfully divide into speech, music, and noise/silence (the accuracy in speech is 91–92%, Music 89–90%, and Noise as 91–92%) by using a hierarchical architecture, which combines hybrid- feature extraction and entropy-based VAD even in the cross-validation evaluation. In addition, we see that the comparison performance is almost robust against different training and the tested dataset.

Secondly, Table 9 illustrates that classification error rates on speech, music, and noise segments are reduced significantly to about a 6% error rate reduction after post-processing. The classification error rates, CER(%) , is defined below as:

(38) $$CER\left(\%\right)=\frac{num.\;of\;falsely\;classfied\;recordings}{total\;num.\;of\;testing\;audio\;recordings}\times 100\%$$

Finally, Table 10 shows that the performance of the proposed audio content classification (ACC) is robust against any variable noise-level conditions under the four types of background noise. Due to the proposed ACC, which is based on a hierarchical approach, it is firstly combined with the two schemes of entropy-based VAD and hybrid-based SMD for classifying the audio content. We can see that the experimental results of the ACC algorithm perform well at four noise types and levels, especially in realistic or poor SNR conditions. The main reason is attributed to the fact that the utilized entropy-based VAD segmentation can also perform successfully in real conditions with variable-noise levels and be excellently applied into the ACC application.

### 5.5. Comparison of Other Classifier Systems

Recently deep convolutional neural networks (deep CNNs) have been very successful at many tasks. The CNNs are designed and exploited to capture audio-related features for the problem of speech and music discrimination [83]. The results of the proposed hierarchical ACC method, along with the other compared methodologies including the deep CNN-based method on the publicly available audio datasets, are presented in Table 11. Our experimental setup was tested on a CPU 2.7 GHz Intel Core i7 for the whole dataset.

The required computational demands are also evaluated in Table 11. We find that the spectrogram-based CNN achieves highest average accuracy with 95.4% under these four datasets. However, the computational time required is also the longest (30.5 min to 36.2 min) to complete the whole evaluation process for these four methods. The computational time includes spectrogram image transform and deep network size for learning features. Deep convolutional neural networks are computationally expensive compared to other systems. They enquire better computing hardware such as GPUs and neuromorphic chips to overcome this drawback. In addition, the CNN has the problem of overfitting and it mostly computationally expensive because it needs to take a large database for training. Compared to the spectrogram-based CNN, the proposed hierarchical ACC system using hybrid feature-based SMD and entropy-based VAD provides a great trade-off in terms of the computing complexity and accuracy. The results show that the execution time of the proposed hierarchical ACC system is almost only half of the spectrogram-based CNN method. Moreover, the average accuracy (with 94.225%) of the proposed hierarchical ACC system is just a little smaller than that of the spectrogram-based CNN method. In actuality, the hierarchical classification has always been one of the great methodologies for audio content analysis. Moreover, a combination of voice activity detection (VAD), speech/music discrimination (SMD), and post-processing is novelty applied into the hierarchical classification. Especially, the voice activity detection (VAD) demonstrates a novel use of entropy. The proposed hierarchical classification system provides a reliable, stable, and low-performance architecture for the audio content analysis.

## 6. Conclusions

In this paper, we presented a new algorithm of audio content classification (ACC) for applications under a variable noise-level environment. A novel hierarchical scene of a three-stage scheme of the proposed ACC algorithm was described in detail for classifying audio stream into speech, music, and background noise. In addition, we introduced the hybrid-based feature, which investigates the use of 1D-subband energy information (1D-SEI) and 2D textural image information (2D-TII) as hybrid features to classify speech or music. It was found that using hybrid-based features can easily discriminate the noisy audio signal into speech and music. Further, the entropy-based VAD segment indeed provides high accuracy for application of the ACC. In summary, we conclude that the proposed ACC based on hybrid features SMD scheme and entropy-based VAD segment can achieve a low error value of below 13% at a low SNR and variable noise-level according to the above experimental results. It was shown that hybrid-based SMD and entropy-based VAD segments can be successfully applied into the system of audio content classification (ACC). The system was tested with different combinations of audio styles and different SNR levels. The experimental evaluations were also performed with real radio recordings from BBC, NHK, and TTV news.

In addition, the proposed hierarchical ACC system was compared with other systems on publicly available audio datasets. This paper proves that the hierarchical classification is one of the great methodologies for audio content analysis. Compared to the spectrogram-based CNN, the proposed hierarchical ACC system using hybrid feature-based SMD and entropy-based VAD can provide a great trade-off in terms of computing complexity and accuracy. Moreover, a combination of voice activity detection (VAD), speech/music discrimination (SMD), and post-processing is a novel idea, applied into the hierarchical classification. Especially, the voice activity detection (VAD) demonstrates a novel use of entropy. The proposed hierarchical classification system provides a reliable, stable, and low-performance architecture for contribution of audio content analysis.

In future work, the proposed ACC approach using hybrid-based manner will be appended to discriminate more audio types with lower SNR levels. In order to apply audio content retrieval, we will also focus on developing an effective scheme.

## Figures and Tables

**Table 1:** Frequency bands limits, in Hz, for Bark scale vs. wavelet scale.

Band Index	Bark Scale		Wavelet Scale		
	Bandlimit	Bandwidth	Bandlimit	Bandwidth	Transform Stage
1	[0–100]	100	[0–125]	125	#5
2	[100–200]	100	[125–250]	125	#5
3	[125–250]	100	[250–375]	125	#5
4	[300–400]	100	[375–500]	125	#5
5	[400–510]	110	[500–625]	125	#5
6	[510–630]	120	[625–750]	125	#5
7	[630–770]	140	[750–875]	125	#5
8	[750–875]	150	[875–1000]	125	#5
9	[920–1080]	160	[1000–1250]	125	#4
10	[1080–1270]	190	[1250–1500]	250	#4
11	[1270–1480]	210	[1500–1750]	250	#4
12	[1480–1720]	240	[1750–2000]	250	#4
13	[1720–2000]	280	[2000–2250]	250	#4
14	[2000–2320]	320	[2250–2500]	250	#4
15	[2320–2700]	380	[2500–3000]	500	#4
16	[2700–3150]	450	[3000–3500]	500	#4
17	[3150–3700]	550	[3500–4000]	500	#4
18	[3700–4400]	700	[4000–5000]	1000	#4
19	[4400–5300]	900	[5000–6000]	1000	#3
20	[5300–6400]	1100	[6000–7000]	1000	#3
21	[6400–7700]	1300	[7000–8000]	1000	#3
22	[7700–9500]	1800	[8000–10,000]	2000	#2
23	[9500–12000]	2500	[10,000–12,000]	2000	#2
24	[12,000–15,500]	3500	[12,000–16,000]	4000	#2

**Table 2:** The evaluation dataset.

#	Duration	Type	Subset
**1**	3840 s	Music, speech	GTZAN Music-Speech [75]
**2**	12 min	Music, speech, silence	Artificial Audio Data
**3**	14 min	Music, Speech, silence, other	Real Radio Recordings
**4**	7 h 37 min	Music, speech, other	LVLib-SMO [12]
**5**	8000 tracks of 30 s	Top 8 genres, balanced with 1000 clips per genre, 1 root genre per clip	FMA-small dataset [76]
**6**	91.6 h	Popular, classical, and jazz music databases	RWC Music Database [77]

**Table 3:** The percentage of distribution between speech, music, and noise.

Arti Num.	Silence	Speech	Music
1	12.54%	42.78%	44.68%
2	9.36%	22.57%	68.07%
3	11.58%	64.38%	24.04%

**Table 4:** The composition of real radio recordings.

Real Num.	Length	Type	Source
1	12 min	TV news clip	NHK and TTV news
2	14 min	Movie clip	BBC radio

**Table 5:** Performance between various VADs under noise conditions.

Method	Acc [%]	Pre [%]	Rec [%]	F1 [%]
Sohn [78]	80.66	85.58	85.92	85.75
Ramirez [79]	89.01	88.81	91.22	90.01
G.729B [80]	56.69	78.31	85.33	81.67
AMR[81]	76.52	70.33	95.86	81.14
Tahmasbi [82]	81.01	85.76	85.33	85.55
**Proposed**	**90.04**	**92.14**	**92.12**	**92.13**

Acc = accuracy, Pre = precision, Rec = recall, F1 = F1-score.

**Table 6:** The classification accuracy under different feature sets.

Feature Set	The Overall Accuracy (%)
**Mel-frequency cepstral coefficient (MFCC)**	88.79
**Spectral centroid (SC)**	91.67
**Zero crossing (ZC)**	76.83
**MFCC + SC + ZC**	92.58
**1D-SEI**	90.51
**2D-TII**	94.35
**Hybrid feature set:** **1D-SEI + 2D-TII**	**96.56**

**Table 7:** Performance of various classifiers with hybrid features on GTZAN dataset.

Classifiers	Speech (%)	Music (%)	Average (%)
SVM (with ERBF kernel)	92.26	90.41	91.33
GMM [47]	90.44	88.58	89.51
k-NN [46]	83.51	82.12	82.81

GMM = Gaussian mixture models; k-NN = k-nearest neighbor.

**Table 8:** Cross-validation evaluation on BBC radio and NHK/TTV news database using the models trained on the GTZAN database.

Models Trained on	BBC Radio Test			NHK/TTV News Test		
	Sp (%)	Mu (%)	No (%)	Sp (%)	Mu (%)	No (%)
GTZAN [75]	91.5	90.4	92.7	92.4	89.2	91.7

Sp (%) = speech (%); Mu (%) = music (%); No (%) = noise/silence (%).

**Table 9:** Comparison with/without post-processing.

With/Without Post-Processing Scheme	Speech CER (%)	Music CER (%)	Noise CER (%)	Overall CER (%)
ACC	17.43	18.62	15.73	17.26
ACC + rule-based post-processing	11.96	12.73	10.89	**11.86**

ACC = audio content classification; CER = classification error rates.

**Table 10:** The classification error rates of the proposed ACC algorithm under four noise types and levels.

Noise Type	SNR (dB)	Dataset					Overall CER (%)
		Arti #01	Arti #02	Arti #03	Real #01	Real #02	
**White Noise**	30	10.38	12.56	11.17	11.89	11.73	11.55
	10	10.49	12.83	11.39	12.28	11.98	11.79
	−5	11.83	14.52	13.58	14.85	13.78	13.71
**Vehicle Noise**	30	9.83	11.29	10.52	11.99	11.84	11.09
	10	10.13	12.03	11.08	12.83	11.94	11.60
	−5	10.52	12.34	11.54	13.69	12.21	12.06
**Factory Noise**	30	7.93	9.27	10.93	10.98	10.23	9.87
	10	8.18	10.12	11.02	12.19	11.82	10.67
	−5	8.34	10.54	11.68	12.43	11.85	10.97
**Babble Noise**	30	11.84	11.48	11.62	12.42	12.94	12.06
	10	12.49	12.69	12.38	13.82	13.39	12.95
	−5	13.53	13.27	13.89	14.59	14.29	13.91
Average							**11.85**

**Table 11:** Comparisons between the proposed hierarchical method and other systems.

Dataset	Proposed Method		k-NN [84]		GMM [85]		Spectrogram-Based CNN [83]	
	Acc (%)	T (min)	Acc (%)	T (min)	Acc (%)	T (min)	Acc (%)	T (min)
GTZAN [75]	93.6	18.6	91.3	20.8	90.4	20.5	94.5	30.5
LVLib-SMO [12]	94.6	18.2	90.8	19.6	88.2	21.1	95.2	33.9
RWC [77]	94.2	19.6	92.2	21.8	89.1	20.8	95.8	35.1
FMA-small [76]	94.5	19.5	92.7	23.1	90.2	22.5	95.7	36.2
AVERAGE	94.225	18.8	91.75	20.73	89.475	20.5	95.4	33.925

T (min) = time in minutes, k-NN = k-nearest neighbor, GMM = Gaussian mixture models.
